# Variant-adapted COVID-19 vaccine boosters enhance humoral immunity and limit IgG4 accumulation in solid cancer patients

**DOI:** 10.3389/fimmu.2025.1699177

**Published:** 2025-12-03

**Authors:** Chiara Piubelli, Matteo Valerio, Donato Zipeto, Elisa Orlandi, Sara Caldrer, Francesco Rizzolo, Katrine Barbero, Elisabetta Vezzelli, Natasha Gianesini, Sonia Zamboni, Natalia Tiberti, Silvia Stefania Longoni, Matteo Verzè, Fabrizio Nicolis, Federico Giovanni Gobbi, Stefania Gori

**Affiliations:** 1 Department of Infectious, Tropical Diseases and Microbiology, IRCCS Sacro Cuore Don Calabria Hospital, Negrar di Valpolicella, Italy; 2 Oncology Department, IRCCS Sacro Cuore Don Calabria Hospital, Negrar di Valpolicella, Italy; 3 Department of Neurosciences, Biomedicine and Movement Sciences, University of Verona, Verona, Italy; 4 Medical Direction Unit, IRCCS Sacro Cuore Don Calabria Hospital, Negrar di Valpolicella, Italy

**Keywords:** solid tumor, SARS-CoV-2, COVID-19, booster vaccination, humoral immuneresponse, infection, immune tolerance

## Abstract

**Background:**

Solid cancer patients are at increased risk of severe COVID-19 and may benefit from repeated booster doses of SARS-CoV-2 mRNA vaccines. Beyond neutralizing antibody titers, recent evidence highlights the induction of spike-specific IgG4 after multiple vaccine doses, a phenomenon potentially linked to immune tolerance.

**Methods:**

We investigated the humoral response following the administration of the fourth and fifth dose of the Comirnaty Omicron XBB.1.5 mRNA vaccine in 48 patients with solid tumors undergoing active or recent anticancer treatment, compared with age-matched controls (n=24). Serum samples were collected before (T1) and three weeks after vaccination (T2). IgG against the spike receptor-binding-domain (IgG-RBD-S), spike-specific IgM (IgM-S), and IgG4 (IgG4-S) were quantified using standardized assays.

**Results:**

Booster vaccination induced a robust increase in neutralizing IgG-RBD-S, with a median 4.6-fold rise at T2 (9568.9 vs. 2086.4 BAU/mL). Notably, high baseline titers at T1 confirmed antibody persistence at about one year after the third dose. IgG-RBD-S levels were higher in patients receiving the fifth compared to the fourth dose, supporting a cumulative dose-dependent effect. IgM-S positivity correlated with significantly stronger neutralizing responses. IgG4-S significantly increased post-vaccination (8.7 to 28.3 ng/mL), but no differences were observed between cancer patients and controls, nor between the fourth and fifth dose, suggesting a lack of further IgG4 accumulation.

**Conclusions:**

Repeated booster doses elicit strong and durable neutralizing antibody responses in solid cancer patients. It seems that variant-adapted formulations may mitigate IgG4 accumulation, thus ideally moderating tolerance associated with IgG4 by introducing antigenic novelty. These findings support continued booster administration and monitoring of humoral responses in oncologic populations.

## 1 Introduction

SARS-CoV-2 has become an endemic virus, present all over the world. Current virus epidemiology does not suggest a potential seasonal trend, but its dynamics seem to be rather influenced by the virus evolution as well as by changes in behavior and public health policy (1). Considering the phenomenon of waning immunity, i.e. a progressive reduction in antibody titers in the months following vaccination or infection (2), and the evolution of viral variants with the potential for immune evasion (3, 4), access to vaccination programs is essential, particularly for fragile populations (such as immunocompromised patients and the elderly). Monitoring COVID-19 vaccination response in fragile individuals remains essential to ensure efficient protection. For solid tumor patients, especially those older or on active chemotherapy, continuous boosters with updated vaccines are strongly recommended to mitigate the risk of the insurgence of severe symptoms or the development of long-COVID sequelae (5–8). With this in mind, every year the Italian Ministry of Health (IMH), in accordance with the most updated WHO, ECDC, EMA and AIFA recommendations, supports the administration of vaccines adapted to the current circulating variant (9–11). For instance, in September 2024 the administration of vaccines adapted for the JN-1 viral variant for the 2024/2025 vaccination campaign has been recommended by the IMH for all persons aged 60 years or older and in several risk categories, including individuals with solid tumors (12). Despite IMH recommendations, data from the 2023–2024 vaccination campaign suggest inadequate uptake, with coverage of 20.8% in the 60–69 age group, 31.9% in the 70–79 age group and 32.4% in the over-80 age group (13).

The benefit of the third booster dose in solid tumor patients has been largely demonstrated (14–16). In a previous study, our group demonstrated that, in a cohort of 273 solid tumor patients at different stages and treated with different anticancer therapies, the persistence of neutralizing antibody and the humoral response after the third dose of the Comirnaty (BioNTech/Pfizer) vaccine was not dependent on either the tumor type, the stage or type of anticancer treatment (17). Only a few direct studies in solid tumor patients after the third dose are available yet (18, 19), but this data, together with trends in other immunocompromised populations, suggest that a 5^TH^ vaccine dose may improve humoral immunity in fragile populations (20). Monitoring antibody titers post-booster can help identify non-responders who may need alternatives (e.g., passive immunization with monoclonal antibodies).

In addition to the anti-spike neutralizing antibodies evaluation, some other parameters should be considered in repeated booster doses vaccination regimens. Recently, it has been demonstrated that the administration of repeated doses of SARS-CoV-2 mRNA-based vaccines results in an increased proportion of spike-specific subtype 4 immunoglobulins (IgG4) production (21–23). Evidence on IgG4 induction following mRNA vaccination remains heterogeneous. Some studies report a stronger class switch toward IgG4 in individuals vaccinated with Comirnaty compared to those receiving Spikevax (Moderna) (24), while others observed higher IgG4 levels and reduced Fc-effector functions in elderly donors immunized with Moderna rather than Pfizer vaccines (25). A third mRNA booster has been associated with increased IgG4 and IgG4-producing memory B cells in recipients of homologous (mRNA/mRNA) regimens, but not in heterologous (adenoviral vector/mRNA) schedules (26). Moreover, immune priming with the first 2–3 mRNA doses appears to drive spike-specific IgG4 development, whereas priming with an adjuvanted protein-based vaccine induces only minimal IgG4 responses, even after further boosters (27).

The immunological significance of IgG4 induction is still a matter of debate, but it is now known that they can lead to an anti-inflammatory response and immune tolerance due to prolonged exposure to high concentrations of the antigen (28). The immune tolerance mechanism is supported by human Interleukin 10 (IL-10)-producing regulatory T cells (Tregs), which are linked to an antigen-specific suppressor function (29, 30). Tregs suppress Th2 cells and their cytokines, reducing the antigen-specific inflammatory response. IL-10 suppresses IgE production and induces IgG4 (29, 30). Literature data reported an increase in IL-10 levels in serum of subjects receiving both primary or booster doses of COVID-19 mRNA vaccine (31). Moreover, in mice it has been shown that prolonged spike receptor-binding domain (RBD) vaccine booster immunization increased the concentration of the immunosuppressive cytokine IL-10 as well as the proportion of IL-10-producing Treg cells (32). Although IgG4 retains neutralizing capability, its anti-inflammatory function is due to its structural attributes, which reduce its ability to mediate Fc-dependent effector functions, such as opsonization by phagocytes, antibody-dependent cellular cytotoxicity (ADCC) by natural killer (NK) cells and antibody-dependent complement deposition (ADCD) (28, 33, 34). The IgG4 Fc domain, in particular, has a reduced affinity for FcγR receptors (34, 35). This reduced affinity is due to the unique characteristic of IgG4 to undergo Fab-arm exchange. This process involves half-molecules of IgG4 randomly combining to form bispecific immunoglobulins with reduced binding activity due to lower valency (36). This attenuation of Fc-mediated functions could potentially predispose individuals to breakthrough infections by dampening part of the adaptive antiviral responses (37, 38). There is a lack of data describing the modulation of IgG4 after repeated doses of the mRNA vaccine for people in frail populations (39). In the elderly, at one month post-third dose, increased levels of IgG4 have been observed in comparison to younger individuals, with a further enhancement after a fifth dose and an association with a reduced capacity of specific serum antibodies to mediate NK cell activation and complement deposition (40). To the best of our knowledge, only one study describes the IgG4 level after repeated vaccination in cancer patients. In this study, performed on pancreatic cancer patients, the authors found higher levels of spike-specific IgG4 in patients who received 3 or more vaccine doses compared to patients vaccinated with 0–2 doses (41). They claimed for a correlation between a poorer pancreatic cancer prognosis and higher level of spike-specific IgG4, but the difference between groups was not statistically significant.

In our study, we analyzed the anti-SARS-CoV-2 humoral response elicited after the administration of 4^TH^ or 5^TH^ dose of the Comirnaty Omicron XBB 1.5 mRNA-based vaccine in a cohort of solid cancer patients. We also evaluated the production of spike-specific IgG4 since this class of immunoglobulins could mediate immune tolerance to SARS-CoV-2 following repeated vaccination boosts.

## 2 Materials and methods

### 2.1 Study design and patient population

Forty-eight adult patients with a solid tumor diagnosis [I, II, III, IV stage, according to the AJCC Cancer Staging Manual classification scale (42)] and active anti-cancer treatment or treatment discontinued for less than 6 months, followed at IRCCS Sacro Cuore Don Calabria Hospital (Negrar di Valpolicella, Verona, Italy), have been recruited at the moment of the booster dose administration of the SARS-CoV-2 vaccine Comirnaty Omicron XBB 1.5 (Pfizer-BioNTech). An age-matched group of subjects without cancer, who received the same dose of vaccine, was used as control (n=24). Serum samples have been longitudinally collected immediately before the administration of the booster doses (T1) and three weeks after the booster doses (T2). Samples were stored at -80°C in the Tropica Biobank (bbmri-eric:ID: IT_1605519998080235) of the IRCCS Sacro Cuore Don Calabria Hospital until use. The following four serology tests were performed on serum samples: a) quantitative measurement of IgG antibodies (including neutralizing antibodies) against the RBD of the S1 subunit of the SARS-CoV-2 spike glycoprotein (IgG-RBD-S); b) measurement of IgG against the nucleocapsid protein (IgG-N); c) measurement of IgM against the spike protein (IgM-S); d) measurement of the spike-specific IgG4 subtype (IgG4-S). Additional data have also been collected on previous COVID-19 or other (anti-influenza, anti-pneumococcus, anti-herpes zoster) vaccinations, SARS-CoV-2 infections and possible vaccination side effects, comorbidities and other pharmacological treatments other than anticancer.

### 2.2 Ethics

The study was conducted in accordance with the Declaration of Helsinki, and approved by the Ethics Committee “Comitato Etico per la Sperimentazione Clinica delle Province di Verona e Rovigo” with the Prot n. 33266 on the 05^TH^ June 2023. Informed consent was obtained from all subjects involved in the study.

### 2.3 Antibody analysis

IgG-RBD-S immunogenicity analysis was conducted using the SARSCoV-2 IgG II Quant assay (Abbott, Ireland) and calibrated to standardized binding antibody Unit/mL (BAU/mL), according to the WHO International Standard (IS 20/136). IgM-S and IgG-N were measured using the SARSCoV-2 IgG-N and the SARS-CoV-2 IgM-S assays (Abbott, Ireland). The automated assays were performed according to the manufacturer’s procedure, using the ARCHITECT i2000 System (Abbott). For IgG-RBD-S, the results were reported according to the following interpretation: BAU/mL<7.1 = negative, BAU/mL≥7.1 = positive. For IgG-N and IgM-S, the results were reported as assay index (S/C) with a positive cut-off ≥1.4 for IgG-N and ≥1 for IgM-S.

IgG4-S serum levels were analyzed by standard ELISA method, using the Anti-SARS-CoV-2 antibody IgG4 Titer serologic Assay Kit (Acro Biosystem, Delaware, USA), following the manufacturer’s instructions. Sample dilution varied from 1:50 to 1:9000. Results were reported as ng/mL, with a positive cut-off >0.1 ng/mL.

### 2.4 Statistical analysis

Statistical analyses were performed using the R software version 4.4.0. Differences in immunologic responses were assessed using the Mann-Whitney U test or the Kruskal-Wallis test for unpaired data at each specified time point, whereas the Wilcoxon signed-rank test was applied to assess differences within groups for paired data at different time points. Pearson’s chi-squared test or Fisher’s exact test were used to compare categorical variables between groups, as appropriate. A p-value < 0.05 was considered statistically significant.

## 3 Results

We characterized the SARS-CoV-2 specific humoral response elicited after repeated booster doses in a cohort of 48 solid tumor patients with active anti-cancer treatment during vaccination, or treatment discontinued for less than 6 months. In particular, we examined the response of a Comirnaty Omicron XBB 1.5 mRNA vaccine (Pfitzer-BioNTech) dose administered to 45 (94%) patients who had previously received at least 3 doses of Comirnaty. Of these patients, 21 (44%) had already received a fourth dose of the Comirnaty Original/Omicron BA.4–5 vaccine. Three patients (6%) received the Comirnaty Omicron XBB 1.5 as a third dose, 24 (50%) as a fourth, 20 (42%) as a fifth and one (2%) as a sixth dose. All had received their previous dose at least 10 months earlier, with an average interval of 14 months for those receiving the 5^TH^ dose and 25 months for those receiving the 4^TH^ dose. Only mild side effects have been reported by 16 patients (33%), with pain at the injection site and malaise as the most frequent ones. No concomitant anti-flu or anti-pneumococcus vaccination was administered. Patients’ characteristics are summarized in **Table 1**. Globally, prostate cancer (23%) and lung cancer (21%) were the most represented tumor types; 52% of patients were at the IV stage and chemotherapy was the most frequent anticancer treatment (50%). Indications about the presence of co-morbidities, other drugs or vaccination administration within the previous 30 days were reported in **Table 1**. Further details are reported in **Supplementary Table S1**. In the age-matched control group, 19 subjects (79%) received the Comirnaty Omicron XBB 1.5 as a fifth dose, 4 (17%) as a fourth and 1 (4%) as a sixth dose. All the subjects received the previous dose about 1 year before. Only mild side effects have been reported by 12 subjects (50%), with pain at the injection site and malaise as the most frequent ones. Fourteen subjects (58%) received anti-flu vaccination within the previous month (**Table 1**).

**Table 1:** Patients’ and control subjects characteristics.

Subject group	Characteristic	Overall	N dose			p-value ^1^
			4^TH^	5^TH^	Other	
Patients		48 (100%)	24 (50%)	20 (42%)	4 (8%)	
	Sex					0.3
	Female	27 (56%)	16 (67%)	9 (45%)	2 (50%)	
	Male	21 (44%)	8 (33%)	11 (55%)	2 (50%)	
	Age at vaccination (years)	73 [66 - 77]	70 [60 - 76]	75 [72 - 78]	66 [62 - 72]	0.12
	Tumor type					>0.9
	Breast	9 (19%)	5 (21%)	4 (20%)	0 (0%)	
	Gastrointestinal	4 (8.3%)	2 (8.3%)	1 (5.0%)	1 (25%)	
	Genitourinary	9 (19%)	5 (21%)	3 (15%)	1 (25%)	
	Lung	10 (21%)	5 (21%)	5 (25%)	0 (0%)	
	Prostate	11 (23%)	5 (21%)	5 (25%)	1 (25%)	
	Other	5 (10%)	2 (8.3%)	2 (10%)	1 (25%)	
	Tumor stage					0.7
	I	3 (6.4%)	1 (4.3%)	2 (10%)	0 (0%)	
	II	6 (13%)	2 (8.7%)	3 (15%)	1 (25%)	
	III	13 (28%)	6 (26%)	5 (25%)	2 (50%)	
	IV	25 (53%)	14 (61%)	10 (50%)	1 (25%)	
	*missing*	1	1	0	0	
	Treatment					0.5
	Chemotherapy	24 (50%)	14 (58%)	8 (40%)	2 (50%)	
	Hormonal therapy	15 (31%)	6 (25%)	8 (40%)	1 (25%)	
	Immunotherapy	7 (15%)	4 (17%)	2 (10%)	1 (25%)	
	Other	2 (4.2%)	0 (0%)	2 (10%)	0 (0%)	
	Other pathologies					0.3
	No	13 (27%)	9 (38%)	3 (15%)	1 (25%)	
	Yes	35 (73%)	15 (63%)	17 (85%)	3 (75%)	
	Other drugs					0.012
	No	12 (25%)	10 (42%)	1 (5.0%)	1 (25%)	
	Yes	36 (75%)	14 (58%)	19 (95%)	3 (75%)	
	Previous vaccination					0.4
	No	35 (73%)	18 (75%)	13 (65%)	4 (100%)	
	Yes	13 (27%)	6 (25%)	7 (35%)	0 (0%)	
Controls		24 (100%)	4 (17%)	19 (79%)	1 (4%)	
	Sex					0.3
	Female	8 (33%)	2 (50%)	5 (26%)	1 (100%)	
	Male	16 (67%)	2 (50%)	14 (74%)	0 (0%)	
	Age at vaccination (years)	68 [61, 81]	59 [57, 61]	71 [65, 82]	61 [-, -]	0.021
	Previous vaccination					>0.9
	No	10 (42%)	2 (50%)	8 (42%)	0 (0%)	
	Yes	14 (58%)	2 (50%)	11 (58%)	1 (100%)	

^1^Kruskal-Wallis rank sum test; Fisher’s exact test.

Descriptive statistic report median [IQR] for continuous variables or n (%) for categorical variables.

We measured the levels of spike-specific IgG (against the receptor binding domain of the spike protein, IgG-RBD-S), IgM (IgM-S), IgG4 (IgG4-S) just before (T1) and 3 weeks after (T2) the vaccine administration. We measured also the nucleocapsid specific IgG (IgG-N), in order to detect a possible previous or concomitant SARS-CoV-2 infection. In fact, despite their reduced durability compared to spike-specific antibodies (43), it is reported that IgG-N are still detectable for more than 15 months after infection (44). We found that 20 (42%) patients had positive IgG-N before and 26 (54%) after the dose administration, indicating a previous and concomitant infection. In total, 17 (65%) subjects reported a previous infection within 3 years, of which only 8 (31%) people reported having had a SARS-CoV-2 infection in the last 15 months. With the exception of one patient who developed pneumonia (infection occurred in 2020, before vaccination), all the other patients reported only mild symptoms. Two patients confirmed to have had COVID-19 early after the dose administration, between T1 and T2. Similarly, in the control group we found that 10 subjects (42%) had positive IgG-N before and 13 (54%) after the dose administration, indicating a previous and concomitant infection. Overall, 10 control subjects (42%) reported a previous infection within the antecedent 3 years, of which 6 (25%) in the last 15 months. Two control subjects confirmed to have had COVID-19 early after the dose administration, between T1 and T2. All reported mild symptoms, and no pneumonia cases were reported.

**Figure 1:**
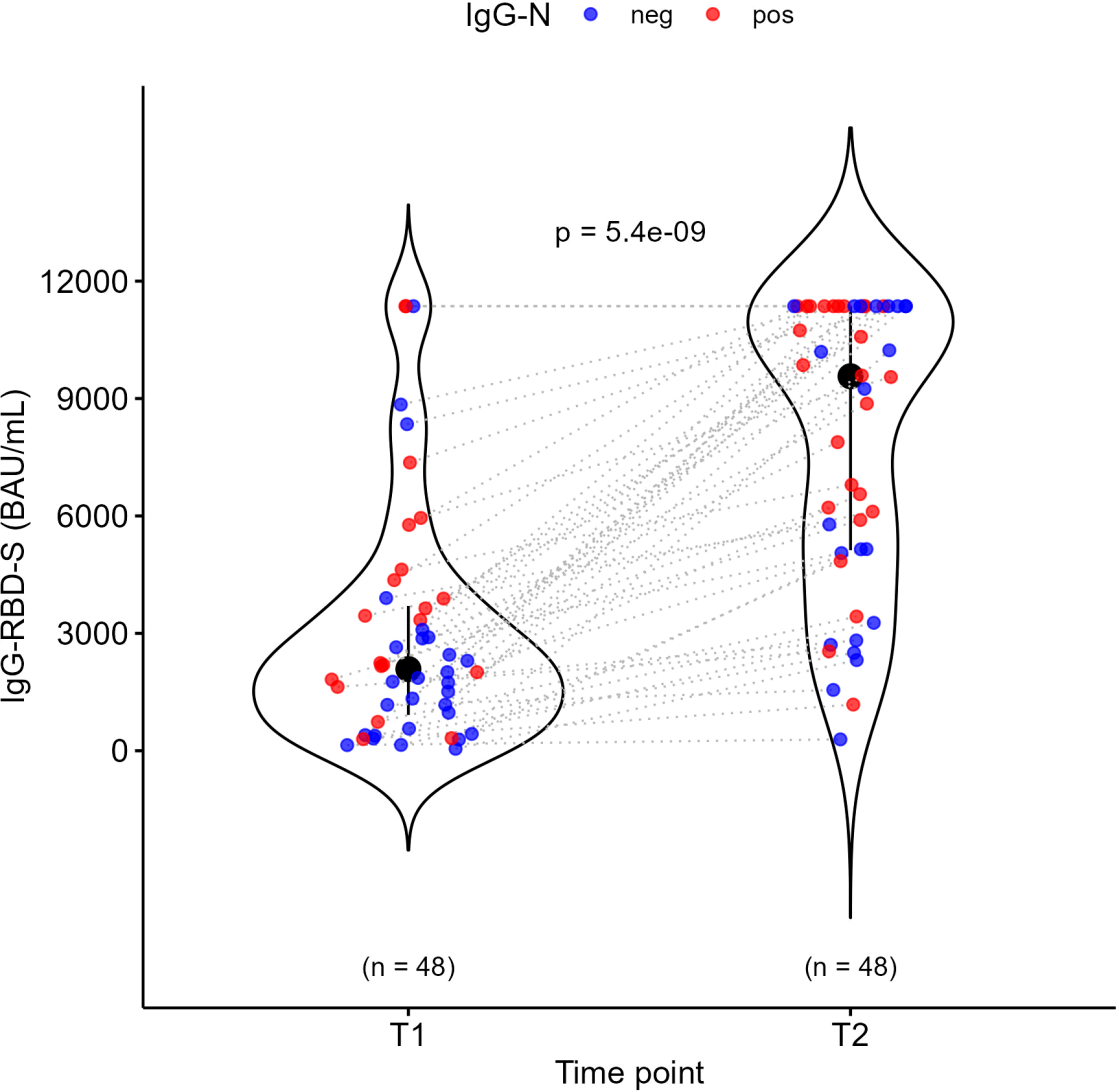
Distribution of IgG-RBD-S antibodies before (T1) and after (T2) the dose administration among the 48 oncologic patients. Wilcoxon signed-rank test was applied to assess differences within groups for paired data at different time points. Patients with positive IgG-N (thus indicating a previous infection) are reported as red dots, while patients with negative IgG-N are reported as blue dots.

**Figure 2:**
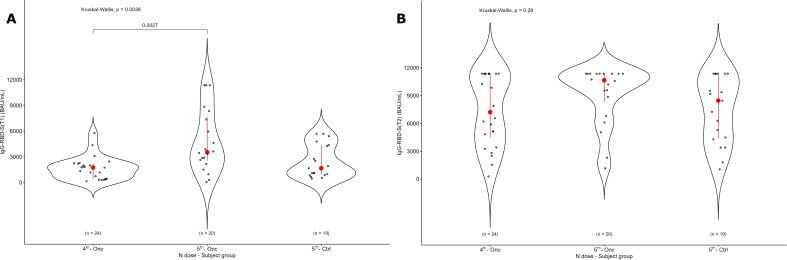
Level of IgG-RBD-S antibodies based on the number of doses. Oncologic patients were divided according to the number of doses, including the Comirnaty Omicron XBB 1.5 they were receiving (4^TH^- Onc and 5^TH^ - Onc, the most represented groups). A control group receiving the 5^TH^ dose is also reported. IgG-RBD-S antibodies levels are shown before (T1, panel A) and after (T2, panel B) the dose administration for the same groups. The p value of Kruskal-Wallis test for unpaired data at a specific time point is reported.

In cancer patients, the median value for IgG-RBD-S antibodies was 2086.4 BAU/mL (IQR: 914.0 – 3701.2) before the dose was administered (T1), increasing by about 4.6-fold to 9568.9 BAU/mL (IQR: 5123.7 – 11360) after the dose was administered (T2) (**Figure 1**). This indicates a strong response to the vaccination. Interestingly, these results showed that a high level of IgG-RBD-S was already present at T1: this could only be partially explained by recent SARS-CoV-2 infections, as inferred from positive IgG-N results. No statistically significant differences were detected in the production of both IgG-RBD-S and IgG-N, neither according to age, sex, tumor type, stage, or anticancer treatment (**Supplementary Figures S1**, **S2**). Comparing the level of neutralizing IgG-RBD-S based on the number of doses (**Table 2**, **Figure 2**), at T1 those receiving the 5^TH^ dose showed higher levels of neutralizing IgG-RBD-S antibodies (p = 0.0027, **Figure 2A**), with no significant difference in the number of previously infected subjects, based on the IgG-N positivity (**Table 3**, Pearson’s Chi-squared test, P value = 0.15). This difference decreased after the dose administration, where only a trend toward an increase was observed between the 4^TH^ and the 5^TH^ dose (the most represented groups), without any statistically significant difference (**Figure 2B**), probably due to the small sample size. An age-matched control group receiving the 5^TH^ dose was used to evaluate the effect of the same vaccination regimen in people without cancer, and we did not detect any significant difference between this group and cancer patients who received the same number of doses (**Table 2**, **Figures 2A, B**).

**Table 2:** Summary table reporting the analyses of all the antibody types, at different doses and timepoints, for both the patients’ and control groups.

Antibody type	Time point T1				Time point T2			
	4^TH^ - Onc N = 24*^1^*	5^TH^ - Onc N = 20*^1^*	5^TH^ - Ctrl N = 19*^1^*	p-value*^2^*	4^TH^ - Onc N = 24*^1^*	5^TH^ - Onc N = 20*^1^*	5^TH^ - Ctrl N = 19*^1^*	p-value*^2^*
IgG-N	0.6 [0.1, 5.4]	1.9 [0.2, 6.0]	1.3 [0.5, 4.3]	0.5	1.3 [0.3, 7.6]	3.4 [0.9, 6.5]	2.2 [0.4, 8.9]	0.7
IgG-RBD-S	1753 [415, 2219]	3544 [2404, 7855]	1672 [881, 4391]	0.004	7221 [4137, 11360]	10660 [7833, 11360]	8464 [4290, 11360]	0.3
IgG4-S	6 [3, 18]	19 [5, 34]	13 [3, 53]	0.12	21 [4, 53]	39 [6, 239]	264 [8, 473]	0.2
IgM-S	0.08 [0.05, 0.25]	0.28 [0.10, 1.44]	0.16 [0.08, 0.33]	0.022	0.5 [0.2, 3.3]	1.8 [0.2, 4.5]	0.4 [0.3, 1.4]	0.7

For the control group, only the most numerous group has been reported (5^TH^ dose). ^1^Median [IQR]; ^2^Kruskal-Wallis rank sum test.

**Table 3:** comparison analysis of the number of IgG-N positive subjects in the 4° and 5° dose groups.

	IgG-N		Total	p-value*^1^*
	neg	pos		
N dose				0.15
4°	16 (67%)	8 (33%)	24 (100%)	
5°	9 (45%)	11 (55%)	20 (100%)	
Total	25 (57%)	19 (43%)	44 (100%)	

*^1^*Pearson’s Chi-squared test.

Data refer to T1 timepoint.

**Figure 3:**
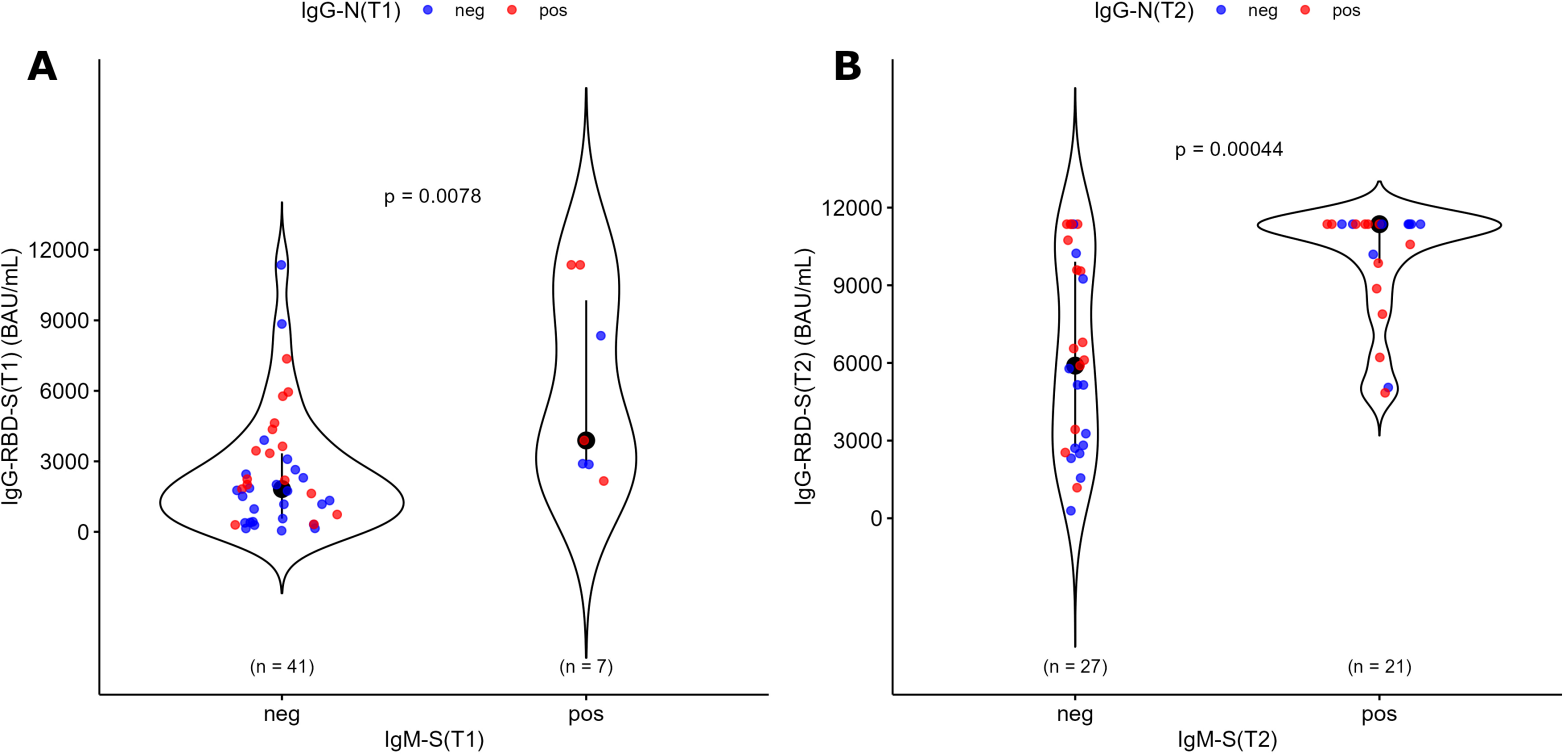
Distribution of IgG-RBD-S antibodies among IgM-S(+) and IgM-S(-) patients before (T1) and after (T2) the dose administration among the 48 oncologic patients. Patients with positive IgG-N (thus indicating a previous infection) are reported as red dots, while patients with negative IgG-N are reported as blue dots. The p value of Mann-Whitney U test for unpaired data at a specific time point is reported.

In our previous works conducted on vaccinated health care workers, we observed an unconventional IgM-S response after vaccination, since only about 50% of naïve subjects produced specific IgM-S (45) and the coordinated expression of IgG-S and IgM-S after vaccination was associated with a significantly more efficient response in neutralizing antibody levels (46, 47). Interestingly, the present study also found that a higher level of IgG-RBD-S was produced in the IgM-S positive (+) population compared to the IgM-S negative population (-) (**Figure 3B**) in the patients’ group, and this statistically significant difference was also confirmed at T1 (**Figure 3A**). IgM-S(+) and IgM-S(-) groups did not differ for the distribution of IgG-N positive subjects, indicating that the results were not influenced by SARS-CoV-2 infections occurrence (**Table 4**, Fisher exact test, P value= 0.4). Similar results were obtained in the control group, but due to the low number of subjects, the statistical significance obtained in the control group could be not reliable (**Supplementary Tables S2**, **S3**). IgM-S values in the patients’ group were not affected by age, sex, tumor type, stage, or anticancer treatment (**Supplementary Figure S3**). We also evaluated the possible influence of other parameters on the IgG-RBD-S levels, such as the presence of comorbidities, the administration of other previous vaccines (e.g. anti-influenza, anti-pneumococcus, anti-herpes zoster) or drugs within 1 month before the COVID-19 vaccination, but we did not find any difference (**Supplementary Figures S4–S6**).

**Table 4:** comparison analysis of the number of previously infected subjects in the IgM(+) and IgM(-) groups.

	IgG-N		Total	p-value*^1^*
	neg	pos		
IgM-S				0.4
neg	25 (61%)	16 (39%)	41 (100%)	
pos	3 (43%)	4 (57%)	7 (100%)	
Total	28 (58%)	20 (42%)	48 (100%)	

*^1^*Fisher’s exact test.

Data refer to T2 timepoint.

**Figure 4:**
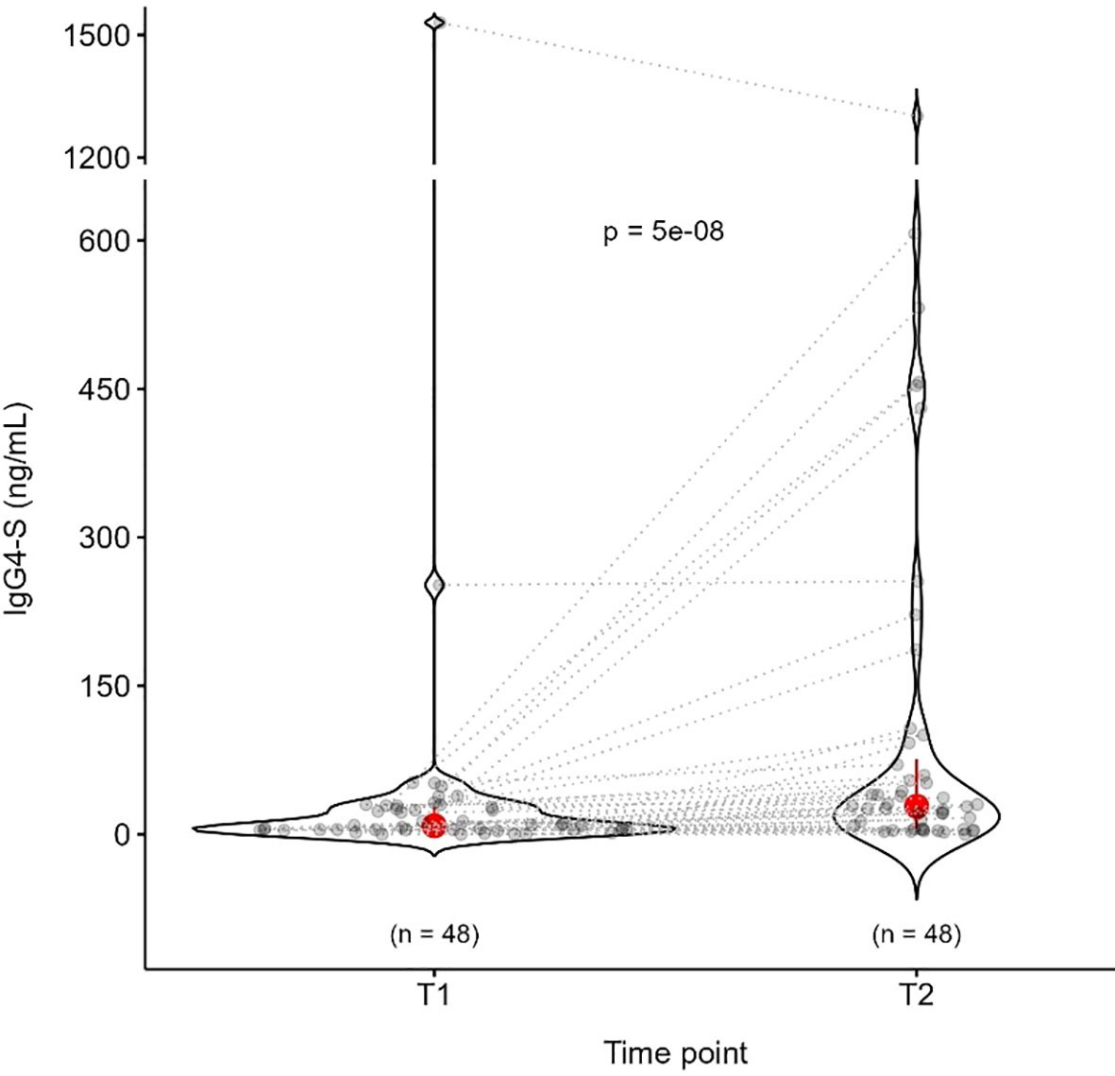
Distribution of IgG4-S antibodies before (T1) and after (T2) the dose administration among the 48 oncologic patients. Wilcoxon signed-rank test was applied to assess differences within groups for paired data at different time points.

**Figure 5:**
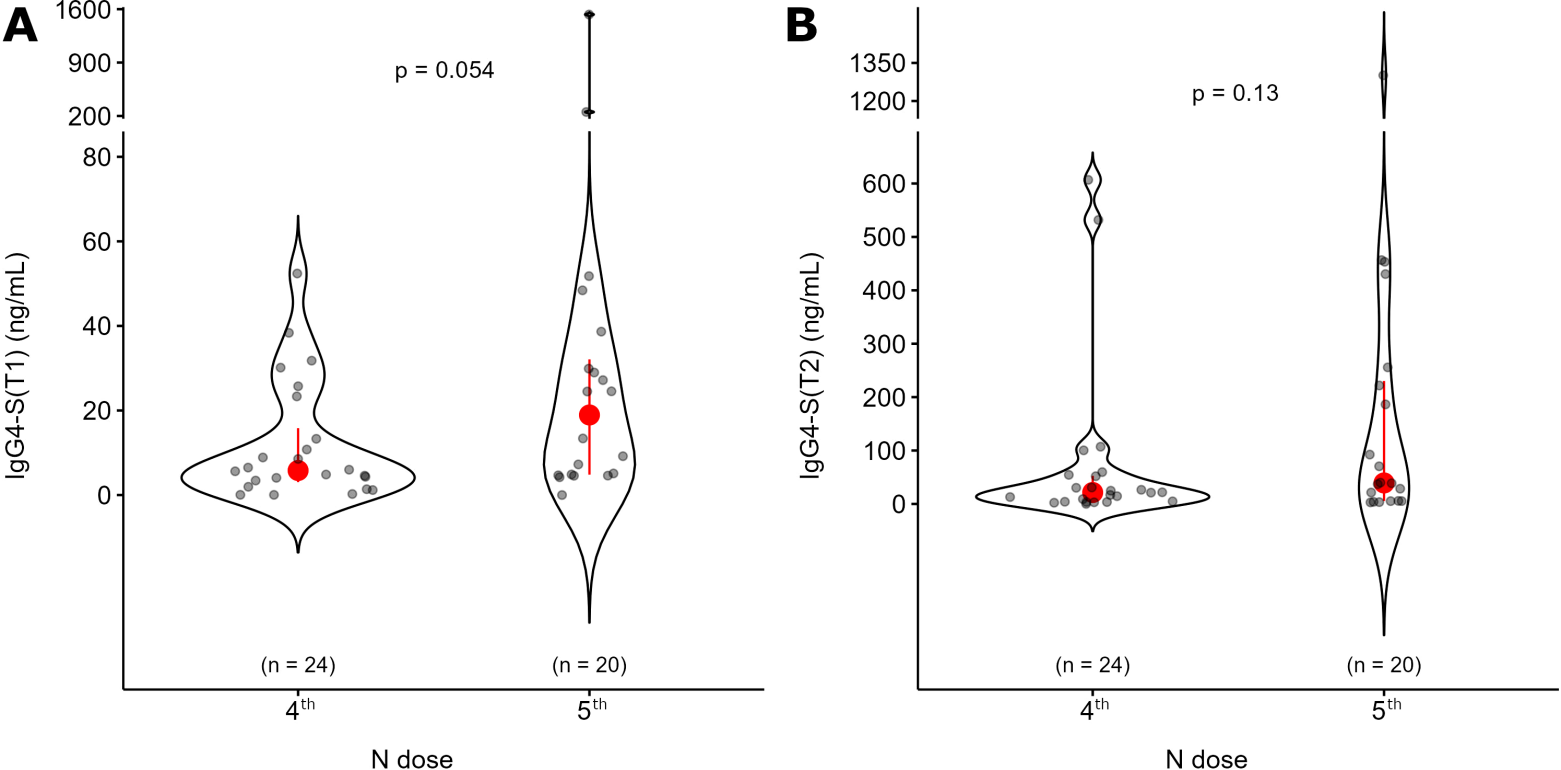
Level of IgG4-S antibodies based on the number of doses. Oncologic patients were divided according to the number of doses, including the Comirnaty Omicron XBB 1.5 they were receiving (4^TH^ - Onc and 5^TH^ - Onc, the most represented groups). IgG4-S antibodies levels are shown before (T1, panel A) and after (T2, panel B) the dose administration for the same groups. The p value of Mann-Whitney U test for unpaired data at a specific time point is reported.

Since the production of spike-specific IgG4 could mediate the development of a possible immune tolerance against SARS-CoV-2, we measured the levels of this subclass of antibodies at T1 and T2. At T1, IgG4-S showed a median value of 8.71 ng/mL, which increased at T2 to 28.32 ng/mL (p = 5E-8, **Figure 4**). Twelve patients (25%) showed higher levels of IgG4-S production (above the third quartile threshold) at T2. Two subjects presented high values of IgG4-S already at T1 (252 and 1530 ng/mL). We did not find any significant difference in the IgG4-S production according to age, sex, tumor type, stage or anticancer treatment (**Supplementary Figure S7**). Comparing the number of doses, we did not find any significant difference between the 4^TH^ and the 5^TH^ doses, either at T1 or T2. (**Figures 5A, B**). No differences were also observed in subjects with positive or negative IgG-N or IgM-S (**Supplementary Figure S8, S9**). We evaluated the possible influence of other co-morbidities, previous vaccinations or other pharmacological treatments on the production of IgG4-S, but no significant differences were detected (**Supplementary Figures S10–S12**).

**Figure 6:**
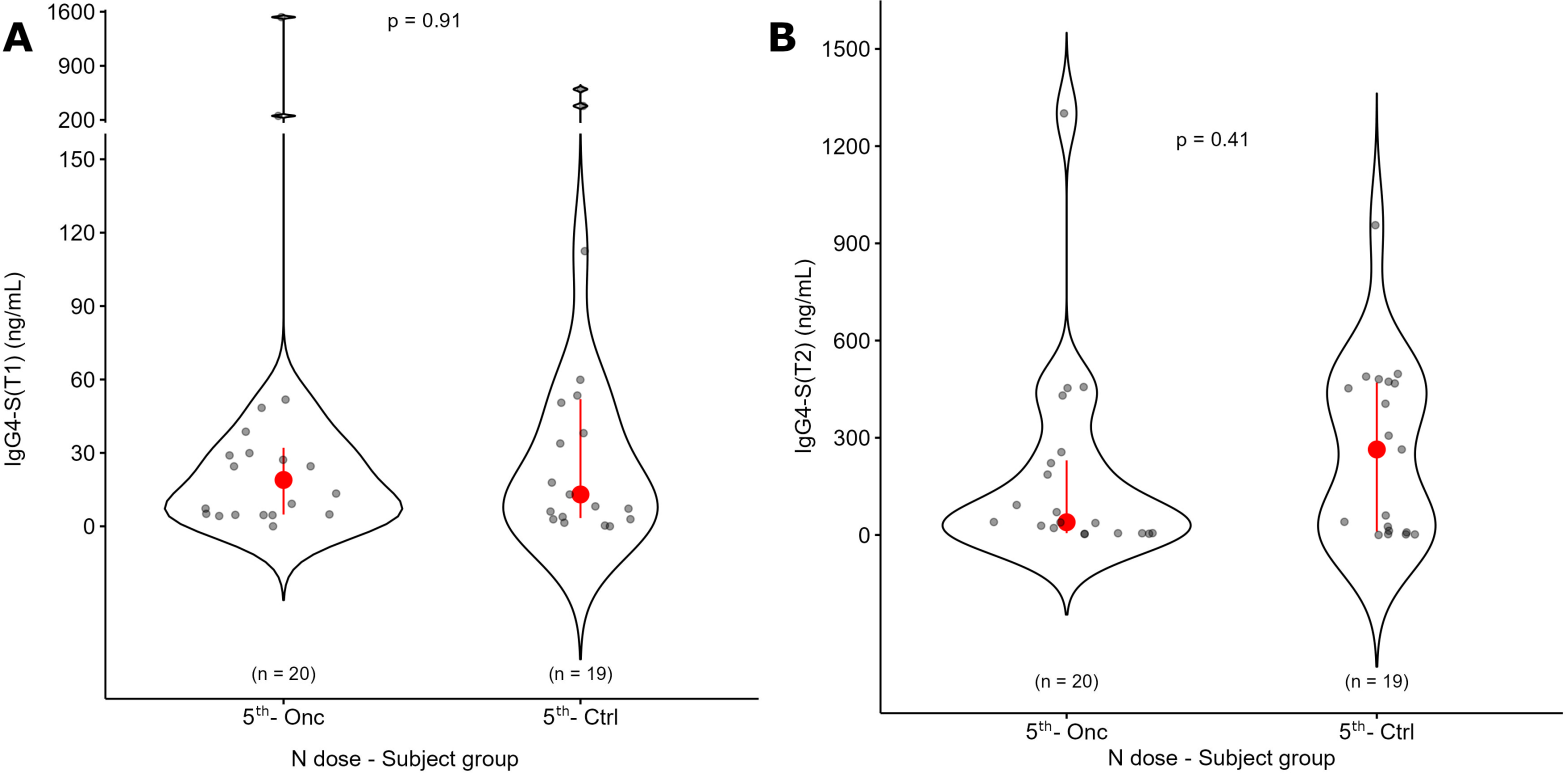
Comparison of IgG4-S antibodies between oncologic patients and a control group. Oncologic patients receiving the 5^TH^ dose were compared with a control group at the same number of doses. IgG4-S antibodies levels are shown before (T1, panel A) and after (T2, panel B) the dose administration for the same groups. The p value of Mann-Whitney U test for unpaired data at a specific time point is reported.

An age-matched control group was used to evaluate the effect of the same vaccination regimen in people without cancer. We did not find a significant difference between the two groups either at T1 or T2 (**Figure 6A, B**).

## 4 Discussion

In this study, we analyzed the humoral immune response elicited by the fourth and fifth doses of the Comirnaty Omicron XBB 1.5 mRNA vaccine in a cohort of solid tumor patients. Our data demonstrate that additional booster doses significantly amplify the neutralizing antibody response (IgG-RBD-S), with a median 4.6-fold increase post-vaccination, independent of tumor type, stage, or anticancer treatment. Notably, considering the average interval between the previous and current booster doses, the persistence of neutralizing antibodies approximately one year after the prior booster dose highlights the durability of humoral immunity in this fragile population, despite the known phenomenon of waning immunity.

**Figure 7:**
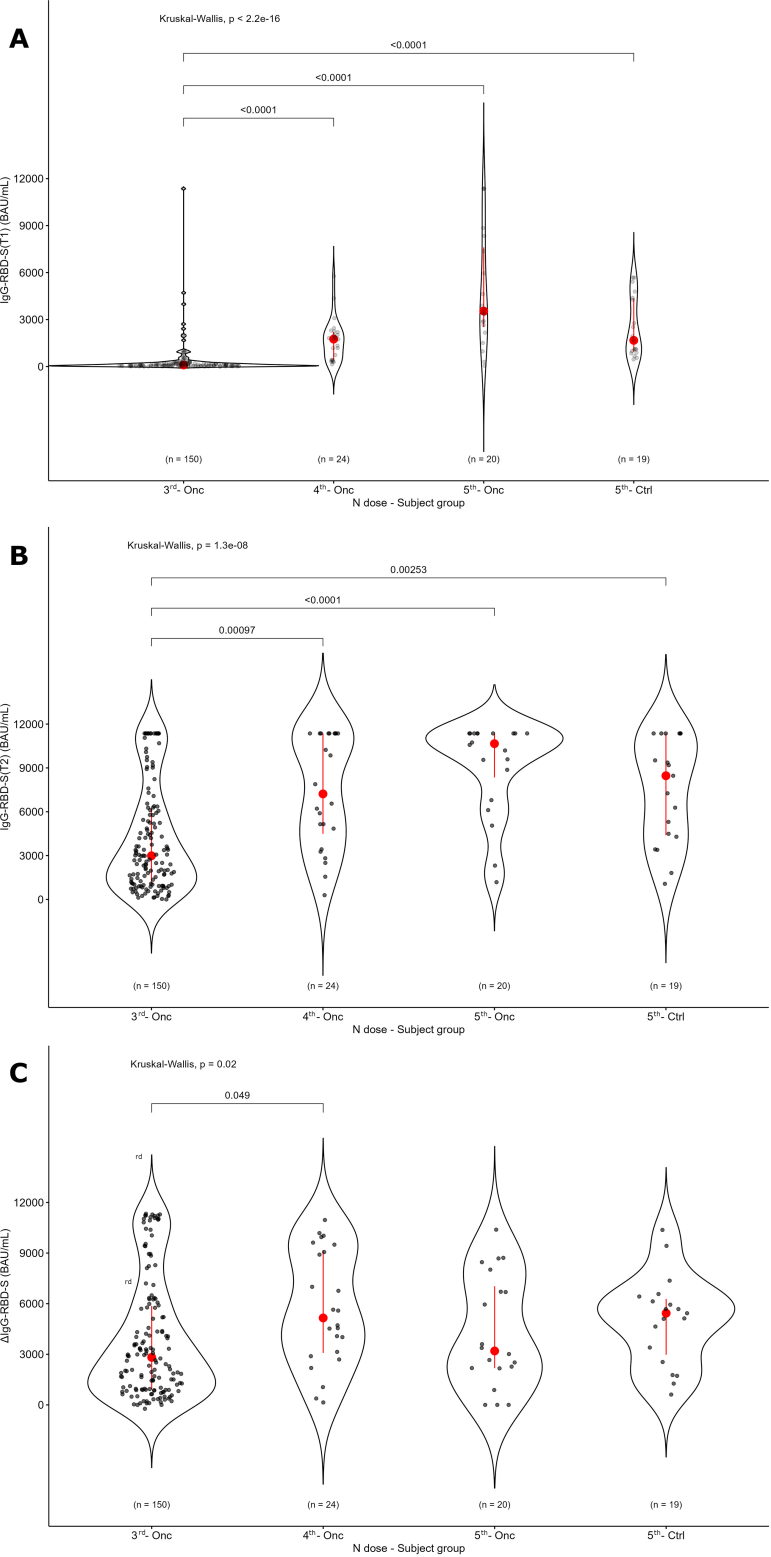
Level of IgG-RBD-S antibodies based on the number of doses. The levels observed in the present study for patients receiving the Comirnaty Omicron XBB 1.5 as the 4^TH^ (4^TH^ - Onc) or the 5^TH^ (5^TH^ - Onc) were compared with data obtained in our previous study on a different cohort of 150 solid tumor patients analyzed before and after the third dose of the Comirnaty Wuhan original vaccine (3^RD^ - Onc) (17). A control group receiving the 5^TH^ dose is also reported. IgG-RBD-S antibodies levels are shown before (T1, panel A) and after (T2, panel B) the dose administration for the same groups. The booster effect based on the antibody level at T1 is reported in panel C (T2-T1). The p value of Kruskal-Wallis test for unpaired data at a specific time point is reported.

To further explore this point, we compared these data with those obtained in our previous study using the same assay and BAU/mL standardized unit, calibrated according to the WHO International Standard, which was applied to a different cohort of 150 solid tumor patients analyzed before and after the third dose of the Comirnaty (Wuhan original) vaccine (17). IgG-RBD-S values before the 3^TH^ dose (median value at T1 = 98.1 BAU/mL, IQR: 38.4 – 210.7) were significantly lower compared to those observed in the current study (median value at T1 for the 4^TH^ dose = 1752.9, IQR: 422.4 – 2207.3; median value at T1 for the 5^TH^ dose = 3544.0, IQR: 2524.1 – 7608.9), confirming that, after the third dose, neutralizing IgG-RBD-S seems to be persistent at least 1 year from the previous vaccination (**Figure 7A**). Comparing the level of neutralizing IgG-RBD-S after the third (data from the previous study (17)), the fourth and the fifth dose (T2), we found a highly statistically significant difference between the third and subsequent doses, with an increase of 1.4 fold after the fourth and 3.6 fold after the fifth dose compared to the third (**Figure 7B**); evaluating the booster effect based on the antibody level at T1, we observed a significant difference between the third and the following doses (**Figure 7C**). These findings confirm that the third booster marks a turning point in the immune response, as from this point onwards neutralizing antibody titers not only persist long term, but also appear to increase proportionally with the number of doses received, highlighting the cumulative effect of repeated boosters. The observed enhancement in IgG-RBD-S titers following repeated booster doses is consistent with prior reports in immunocompromised populations, reinforcing the critical role of booster vaccinations in maintaining an adequate and durable immune protection against SARS-CoV-2 (18, 19).

Interestingly, patients producing specific IgM-S after boost vaccination showed significantly higher IgG-RBD-S levels post-booster. These data are in line with our previous studies conducted on a cohort of about 2000 healthcare workers, where we observed that the development of IgM-S in response to the first two vaccination doses was associated with higher IgG-RBD-S levels at short and long follow-up and persisted even after the third dose (46, 47). Thus, our data suggest that in cancer patients too, elicitation of IgM-S following vaccination should be considered an indicator of a stronger neutralizing response. Therefore, IgM-S production could be used as a biomarker of a more efficient immune response after vaccination.

Regarding IgG4 dynamics, we confirmed an increase in spike-specific IgG4 levels following repeated vaccine doses. This finding aligns with recent studies indicating that repeated mRNA vaccine exposure promotes IgG4 subclass switching, a phenomenon associated with immune regulation and tolerance (28, 33). Tam et al. (48) recently highlighted that IgG4 production is affected by the number of vaccine doses received, the interval between them and SARS-CoV-2 infections. Through functional studies, they demonstrated that, while IgG4 poorly activates ADCC, ADCP and opsonization by phagocytes, it is capable of equivalent and potent neutralization to IgG1. Notably, IgG4 exhibits anti-inflammatory properties only when it competes directly with an activating IgG1 for binding; otherwise, it is functionally silent (48). Importantly, in our cohort, the increase in IgG4-S production was not associated with tumor type, stage, treatment regimen, or other vaccination or pharmacological treatments, suggesting that the shift in IgG4-S is predominantly driven by antigenic exposure rather than clinical characteristics in this patient group. In addition, no significant differences in IgG4-S levels were observed between patients with solid tumors and age-matched healthy controls, indicating that the IgG4-S response elicited by booster vaccination in these oncologic patients is similar to that observed in the general population. Therefore, it can be concluded that the oncologic condition or anticancer treatments do not amplify this phenomenon.

Crucially, we did not observe a significant difference in IgG4-S levels between the fourth and fifth booster doses. This observation suggests that the antigenic composition of the vaccine, particularly the exclusion of the ancestral Wuhan-Hu-1 spike sequence in updated formulations (e.g., Omicron XBB.1.5), may limit further IgG4-S accumulation despite repeated antigen exposures. In fact, recent studies on bivalent mRNA boosting, which still included the original Wuhan antigen, have shown that it produced a relevant IgG4 response with poor Fc functional activity (49). It is conceivable that variant-adapted vaccines reduce the likelihood of inducing immune tolerance by introducing antigenic novelty (50); however, this hypothesis warrants confirmation through longitudinal investigations, including Fc-dependent functional activity studies, such as opsonization by phagocytes, ADCC and ADCP assays.

From a clinical perspective, these results indicate that the humoral response activated by the booster doses after the third is long-lasting and that the concerns regarding IgG4-associated immune tolerance could probably be mitigated by antigen variation in updated vaccines.

Our study is limited by the relatively small sample size. Although supported by literature data, the absence of a functional assessment of Fc-mediated antibody effector functions (e.g., ADCC, ADCP, opsonization assays) makes the hypothesis on immunological tolerance in our patients’ population only theoretical and should be confirmed by experimental data. Furthermore, the lack of longitudinal follow-up beyond three weeks post-booster precludes conclusions regarding the durability of the observed responses. Further studies involving extended observation periods and larger cohorts are needed to determine the clinical implications of IgG4 modulation in cancer patients more precisely.

## 5 Conclusions

In summary, our study demonstrates that repeated mRNA COVID-19 boosters in solid cancer patients elicit strong and persistent neutralizing antibody responses, with titers increasing in proportion to the number of administered doses, regardless of tumor type, disease stage, or treatment regimen. Importantly, the induction of IgG4 following booster vaccination was comparable between cancer patients and aged-matched controls, indicating that the oncologic status does not exacerbate this phenomenon.

Although an increase in spike-specific IgG4 antibodies was observed following multiple immunizations, the levels seem to be mitigated in the last doses in which the Wuhan original antigen was absent. These results support the continued administration of variant-adapted boosters in oncological populations to maintain effective immune protection, and highlight the importance of monitoring the insurgence of vaccine-induced immune tolerance. Expanding vaccine coverage in fragile cohorts remains a priority to mitigate severe COVID-19 outcomes and the spread of emerging variants.

## Data Availability

The datasets presented in this study can be found in online repositories. The names of the repository/repositories and accession number(s) can be found below: The dataset for this study is available upon request in Zenodo at the following link https://doi.org/10.5281/zenodo.17053315.
