# Supplementary material for: Variant-adapted COVID-19 vaccine boosters enhance humoral immunity and limit IgG4 accumulation in solid cancer patients
Source: Front Immunol. 2025 Dec 3;16:1699177. doi: 10.3389/fimmu.2025.1699177 (PMC12708278; doi:10.3389/fimmu.2025.1699177)
Supplement: Supplementary file 1 [file DataSheet1.docx]

Supplementary Material

# Supplementary Tables

| **Supplementary Table S1: additional details about cancer patients’ cohort.**  **Other tumor type** | **n** |
| --- | --- |
| Pancreas | 1 |
| Uterus | 1 |
| Liver | 2 |
| missing | 1 |
|  |  |
| **Other pathologies in addition to cancer** | **n** |
| autoimmune disease | 2 |
| Chronic pulmonary disease | 2 |
| Liver disease | 2 |
| hypetension | 25 |
| cardiac disease | 10 |
| diabetes | 8 |
| peripheral vascular disease | 1 |
|  |  |
| **Other drugs in addition to anticancer treatment** | **n** |
| antidiabetic | 8 |
| anticoagulant | 4 |
| antiaggregant | 4 |
| antihypertensive | 25 |
| nonsteroidal anti-inflammatory | 1 |
| corticosteroids | 5 |
| antihistamines | 2 |
| valproic acid | 1 |
| thyroid hormone | 1 |
|  |  |
| **Other vaccination before COVID-19 dose** | **n** |
| anti-flu | 11 |
| anti-pneuomococcus | 1 |
| missing | 1 |

**Supplementary Table S2: Distribution of IgG-RBD-S antibodies among IgM-S(+) and IgM-S(-) patients before the dose administration (T1) among the 24 control subjects and** **comparison analysis of the number of previously infected subjects (IgG-N positive) in the IgM(+) and IgM(-) groups.** Data refer to T1 timepoint.

|  | IgM-S | |  |
| --- | --- | --- | --- |
|  | **neg** N = 23*^1^* | **pos** N = 1*^1^* | **p-value** |
| IgG-RBD-S | 1125 [839, 4266] | 5419 [-, -] | - |
| *^1^* Median [Q1, Q3] | | | |

|  | **IgG-N** | | **Total** | **p-value***^1^* |
| --- | --- | --- | --- | --- |
|  | neg | pos |  |  |
| **IgM-S** |  |  |  | 0.4 |
| neg | 14 (61%) | 9 (39%) | 23 (100%) |  |
| pos | 0 (0%) | 1 (100%) | 1 (100%) |  |
| **Total** | 14 (58%) | 10 (42%) | 24 (100%) |  |
| *^1^* Fisher’s exact test | | | | |

**Supplementary Table S3: Distribution of IgG-RBD-S antibodies among IgM-S(+) and IgM-S(-) patients after the dose administration (T2) among the 24 control subjects and** **comparison analysis of the number of previously infected subjects (IgG-N positive) in the IgM(+) and IgM(-) groups.** Data refer to T2 timepoint.

|  | IgM-S | |  |
| --- | --- | --- | --- |
|  | **neg** N = 18*^1^* | **pos** N = 6*^1^* | **p-value***^2^* |
| IgG-RBD-S | 6771 [3423, 9518] | 11360 [11360, 11360] | 0.022 |
| *^1^* Median [Q1, Q3], *^2^* Wilcoxon rank sum test | | | |

|  | **IgG-N** | | **Total** | **p-value***^1^* |
| --- | --- | --- | --- | --- |
|  | neg | pos |  |  |
| **IgM-S** |  |  |  | 0.6 |
| neg | 9 (50%) | 9 (50%) | 18 (100%) |  |
| pos | 2 (33%) | 4 (67%) | 6 (100%) |  |
| **Total** | 11 (46%) | 13 (54%) | 24 (100%) |  |
| *^1^* Fisher’s exact test | | | | |

# Supplementary Figures

**Supplementary Figure S1.** **IgG-RBD-S values in solid cancer patients according to different parameters.** The considered parameters are: age (<70, ≥70 years), sex (male, female), tumor type, cancer stage, anticancer treatment type. Mann-Whitney U test or Kruskal-Wallis rank sum test results are reported for each considered parameters. A p-value < 0.05 was considered as statistically significant.


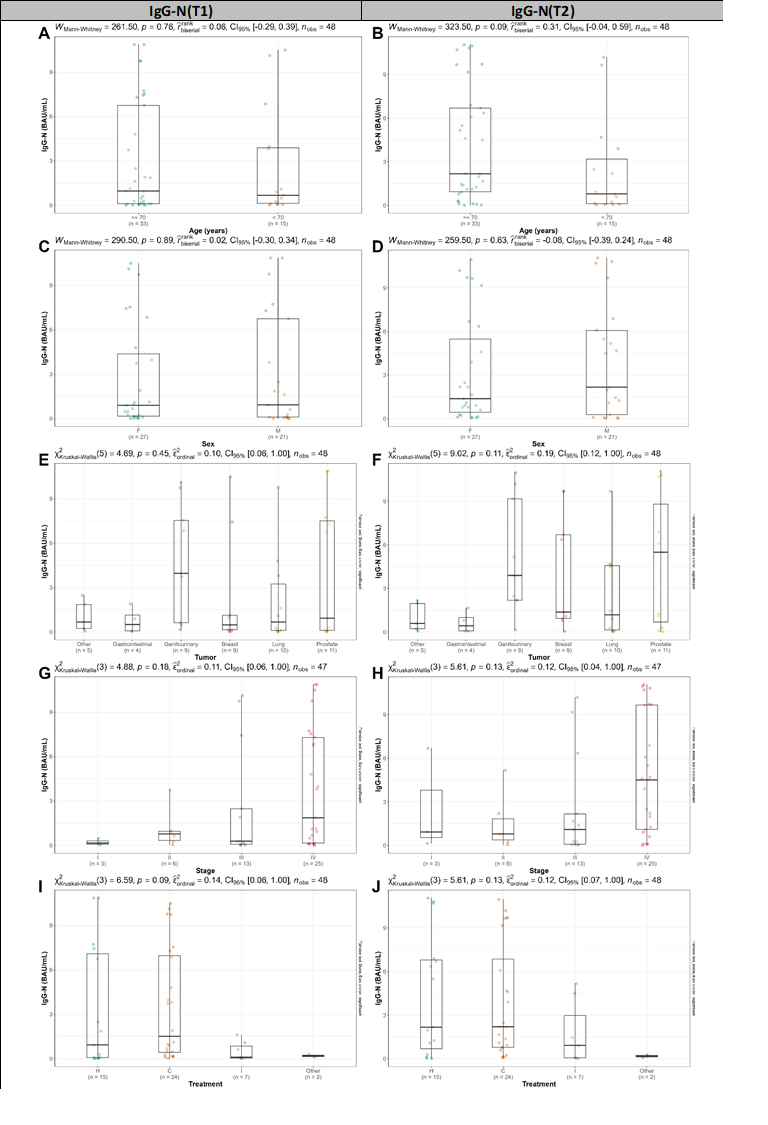


**Supplementary Figure S2.** **IgG-N values in solid cancer patients according to different parameters.** The considered parameters are: age (<70, ≥70 years), sex (male, female), tumor type, cancer stage, anticancer treatment type. Mann-Whitney U test or Kruskal-Wallis rank sum test results are reported for each considered parameters. A p-value < 0.05 was considered as statistically significant.

**Supplementary Figure S3.** **IgM-S values in solid cancer patients according to different parameters.** The considered parameters are: age (<70, ≥70 years), sex (male, female), tumor type, cancer stage, anticancer treatment type. Mann-Whitney U test or Kruskal-Wallis rank sum test results are reported for each considered parameters. A p-value < 0.05 was considered as statistically significant.

**Supplementary Figure S4.** **IgG-RBD-S values in solid cancer patients according to the presence of other pathologies in addition to cancer.** Data are shown for both timepoints (T1 and T2). Mann-Whitney U test results are reported for each timepoint. A p-value < 0.05 was considered as statistically significant.

**Supplementary Figure S5.** **IgG-RBD-S values in solid cancer patients according to the administration of other vaccines prior to COVID-19 vaccination.** Data are shown for both timepoints (T1 and T2). Mann-Whitney U test results are reported for each timepoint. A p-value < 0.05 was considered as statistically significant.

**Supplementary Figure S6.** **IgG-RBD-S values in solid cancer patients according to the administration of other drugs in addition to the anticancer treatment.** Data are shown for both timepoints (T1 and T2). Mann-Whitney U test results are reported for each timepoint. A p-value < 0.05 was considered as statistically significant.

**Supplementary Figure S7.** **IgG4-S values in solid cancer patients according to different parameters.** The considered parameters are: age (<70, ≥70 years), sex (male, female), tumor type, cancer stage, anticancer treatment type. Mann-Whitney U test or Kruskal-Wallis rank sum test results are reported for each considered parameters. A p-value < 0.05 was considered as statistically significant.

**Supplementary Figure S8.** **IgG4-S values in solid cancer patients according to IgG-N positivity.** IgG-N positive values were considered for the evaluation of previous SARS-CoV-2 infection. Data are shown for both timepoints (T1 and T2). Mann-Whitney U test results are reported for each considered parameters. A p-value < 0.05 was considered as statistically significant.

**Supplementary Figure S9.** **IgG4-S values in solid cancer patients according to IgM-S positivity.** Data are shown for both timepoints (T1 and T2). Mann-Whitney U test results are reported for each considered parameters. A p-value < 0.05 was considered as statistically significant.

**Supplementary Figure S10.** **IgG4-S values in solid cancer patients according to the presence of other pathologies in addition to cancer.** Data are shown for both timepoints (T1 and T2). Mann-Whitney U test results are reported for each timepoint. A p-value < 0.05 was considered as statistically significant.

**Supplementary Figure S11.** **IgG4-S values in solid cancer patients according to the administration of other vaccines prior to COVID-19 vaccination.** Data are shown for both timepoints (T1 and T2). Mann-Whitney U test results are reported for each timepoint. A p-value < 0.05 was considered as statistically significant.

**Supplementary Figure S12.** **IgG4-S values in solid cancer patients according to the administration of other drugs in addition to the anticancer treatment.** Data are shown for both timepoints (T1 and T2). Mann-Whitney U test results are reported for each timepoint. A p-value < 0.05 was considered as statistically significant.
